# Regulated protein aggregation: stress granules and neurodegeneration

**DOI:** 10.1186/1750-1326-7-56

**Published:** 2012-11-20

**Authors:** Benjamin Wolozin

**Affiliations:** 1 Departments of Pharmacology and Neurology, Boston University School of Medicine, 72 East Concord St., R614, Boston, MA, 02118-2526, USA

**Keywords:** Stress granule, TIA-1, TIAR, TTP, G3BP, Prion protein, Microtubule associated protein tau, TDP-43, FUS, FMRP, Prion protein protein synthesis, RNA translation, Alzheimer’s disease, Amyotrophic lateral sclerosis, Motor neuron disease, Frontotemporal dementia

## Abstract

The protein aggregation that occurs in neurodegenerative diseases is classically thought to occur as an undesirable, nonfunctional byproduct of protein misfolding. This model contrasts with the biology of RNA binding proteins, many of which are linked to neurodegenerative diseases. RNA binding proteins use protein aggregation as part of a normal regulated, physiological mechanism controlling protein synthesis. The process of regulated protein aggregation is most evident in formation of stress granules. Stress granules assemble when RNA binding proteins aggregate through their glycine rich domains. Stress granules function to sequester, silence and/or degrade RNA transcripts as part of a mechanism that adapts patterns of local RNA translation to facilitate the stress response. Aggregation of RNA binding proteins is reversible and is tightly regulated through pathways, such as phosphorylation of elongation initiation factor 2α. Microtubule associated protein tau also appears to regulate stress granule formation. Conversely, stress granule formation stimulates pathological changes associated with tau. In this review, I propose that the aggregation of many pathological, intracellular proteins, including TDP-43, FUS or tau, proceeds through the stress granule pathway. Mutations in genes coding for stress granule associated proteins or prolonged physiological stress, lead to enhanced stress granule formation, which accelerates the pathophysiology of protein aggregation in neurodegenerative diseases. Over-active stress granule formation could act to sequester functional RNA binding proteins and/or interfere with mRNA transport and translation, each of which might potentiate neurodegeneration. The reversibility of the stress granule pathway also offers novel opportunities to stimulate endogenous biochemical pathways to disaggregate these pathological stress granules, and perhaps delay the progression of disease.

## Introduction

The purpose of the review is to provide a new perspective on the role of protein aggregation in neurodegenerative disease. This perspective seeks to incorporate the concept of regulated protein aggregation into the pathophysiology of neurodegenerative diseases. Highlighting the role of regulated protein aggregation in disease pathology provides a biological context for understanding how the process of pathological protein aggregation in disease might evolve, identifies a broad range of proteins that co-aggregate during disease (beyond the classic insoluble protein disease markers), illuminates signaling cascades regulating many of these aggregation processes and provides potential pathways for therapeutic intervention.

**Figure 1:**
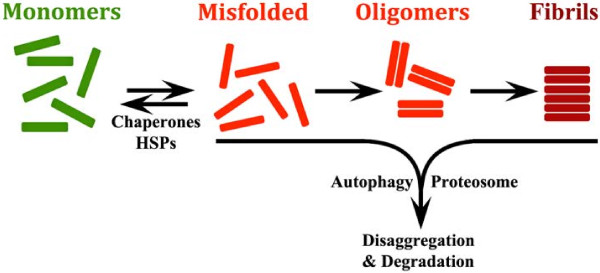
**The conventional model for degenerative disease based on mass action and hydrophobic interactions.** Monomeric proteins randomly misfold. The chaperone system, including heat shock proteins (HSPs), can reverse the misfolding, and produce normal, functional proteins. However, the misfolded proteins are prone to random oligomerization, and evidence suggests that the resulting oligomers can be toxic. The oligomers aggregate further to form fibrillar aggregates. In each case formation of the misfolded proteins, oligomers and fibrils are considered to lack normal biological functions. These oligomers and fibrillar aggregates can be removed by degradation, which occurs through the actions of the autophagic system and the ubiquitin proteasomal system. Increasing evidence suggests that autophagy is the predominant mechanism of degradation in diseases such as Alzheimer’s disease and Parkinson’s disease [3].

The classic model for pathological protein aggregation in neurodegenerative diseases is based on mass action and energy minimization, as elegantly proposed by Dobson and Lansbury (Figure 1) [1,2]. In this model, aggregation prone proteins are initially present in a cell as monomers. Some of these proteins randomly misfold in a process that is thought to be devoid of biological function. The misfolded proteins oligomerize, and aggregate further to form fibrils. In each case, these processes are thought to occur as undesirable, nonfunctional biological events. The rate of aggregation of these proteins depends on the amount of starting material (monomeric protein) and the propensity of the protein to aggregate. More starting material leads to more oligomerization by the law of mass action. Greater hydrophobicity also increases the propensity of a protein to oligomerize, due to a greater the need for energy minimization of the hydrophobic interactions. Oligomer and fibril accumulation are minimized by the actions multiple protein chaperones, such as heat shock proteins, as well as by actions of the ubiquitin proteasomal and autophagic systems. Which act to degrade the accumulating oligomers and fibrils [3,4]. Despite the elegant complexity of chaperones, the proteasomal system and autophagy, the fundamental concept remains; protein aggregation largely occurs as an unwanted chemical reaction driven by concentration gradients and hydrophobicity. These unwanted oligomers and fibrils appear to cause toxicity, which elicits cell death and inflammation. An example of the broader neurodegeneration hypotheses is the amyloid cascade hypothesis, which links all of this together in a recursive cycle [5].

## RNA binding proteins: biology and contributions to neurodegenerative diseases

**Figure 2:**
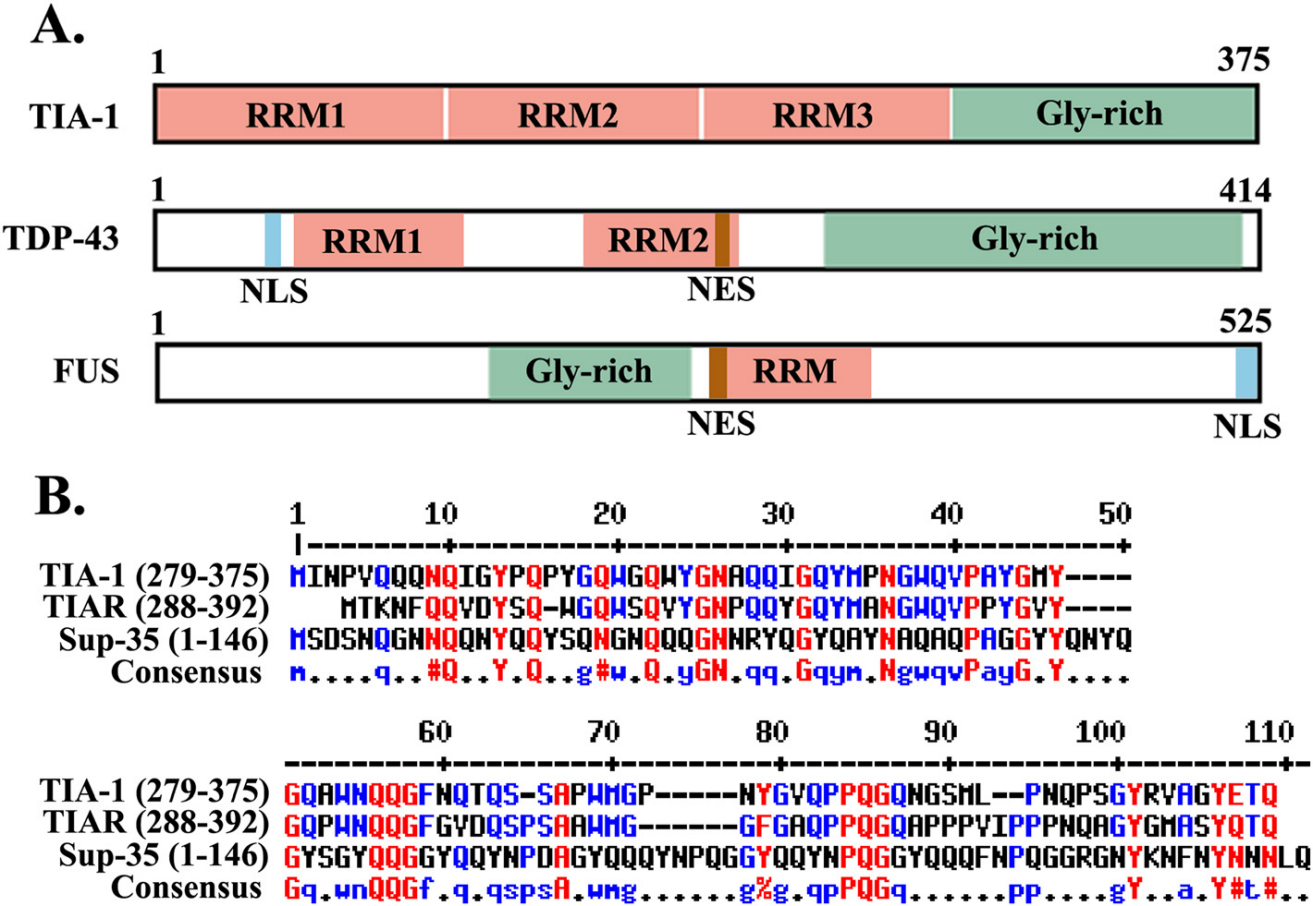
**Structures of RNA binding proteins.A**) RNA binding proteins, such as TIA-1, TDP-43 and FUS, contain RNA-recognition motifs (RRM), which bind RNA and Glycine rich (Gly-rich) domains that mediate protein aggregation. TDP-43 and FUS contain discrete nuclear localization and nuclear export signals (NLS, NES), while nuclear localization of TIA-1 does not appear to localize to particular domains [11,12]. **B**) Alignment of glycine rich domains of TIA-1, TIAR and Sup-35. The alignment was performed using the “multalin” program (http://multalin.toulouse.inra.fr). In the consensus sequence, the red letters correspond to homologous amino acids, where the # symbol is used to convey imperfect homologies, and the blue letters refer to partial homology.

The classical process of pathological protein aggregation contrasts with the tightly regulated and reversible process of aggregation that occurs as an intrinsic aspect of the biology of RNA binding proteins. RNA binding proteins have gained attention recently because of the large number of these proteins that are mutated in familial forms of motor neuron diseases. Mutations in RNA binding proteins such as Tar DNA binding protein-43 (TDP-43), Fused in sarcoma (FUS), survival of motor neuron (SMN1), ataxin-2 (ATX2), optineurin (OPT) and angiogenenin (ANG) all cause motor neuron diseases. The roughly 800 proteins in this family exhibit conserved domains structures, and related functions (Figure 2). These RNA binding proteins generally contain two types of conserved domains: glycine rich domains and RNA recognition motifs (RRM). The glycine rich domain is hydrophobic and mediates the reversible aggregation of these proteins; for some RNA binding proteins, such as TIA-1, but not TDP-43, the glycine rich domain shares homology with the yeast prion protein, Sup35 (Figure 2); homology between Sup-35 and TDP-43 is much weaker [6,7]. The RRMs have broad specificity, but differ in the spectrum of transcripts bound. For instance, T-intracellular antigen-1 (TIA-1) recognizes transcripts with a uracyl-rich motif with a 30–37 nucleotide long bipartite motif [8]. TIA1 cytotoxic granule-associated RNA binding protein-like 1 (TIAR) binds transcripts with a 28–32 nucleotide long stem loop element [9]. The Hu family of RNA binding proteins bind transcripts with a 17–20 nucleotide segment rich in uracyls [10].

The concept of reversible, regulated protein aggregation is central to the hypothesis proposed in this article and demands clarification at the outset. The concept of beneficial misfolding was perhaps first examined in yeast, where the elongation initiation factor Sup35 was shown to misfold in response to environmental (nutritional) stress, and alter the synthesis of proteins in a manner that promotes yeast survival [7]. Sup35 misfolding is stable, is transmitted among yeast, and analogous to the biology of prions [7]. Glycine rich domains in Sup35 mediate the misfolding and also give rise to insoluble protein aggregates, much like amyloidogenic proteins that aggregate in neurodegenerative diseases [13]. The homology between glycine rich domains of some mammalian RNA binding proteins, such as TIA-1, and Sup35 highlights a putative role of regulated protein aggregation in the biology of RNA binding proteins (Figure 2). The glycine rich domain of other RNA binding proteins, such as TDP-43, are not homologous to Sup35, but are capable of interacting with Sup35, which emphasizes the biological relatedness of these proteins [14]. The response of many of these RNA binding proteins to stress resembles that of Sup35 in that they aggregate to form insoluble macromolecular structures, composed of RNA binding proteins and mRNA, termed stress granules (see below for further discussion of stress granules) [15]. The resulting aggregates are detergent insoluble, and can be isolated through classic methods used to isolate insoluble proteins aggregates present in brain tissues of subjects with neurodegenerative diseases [16,17].

**Figure 3:**
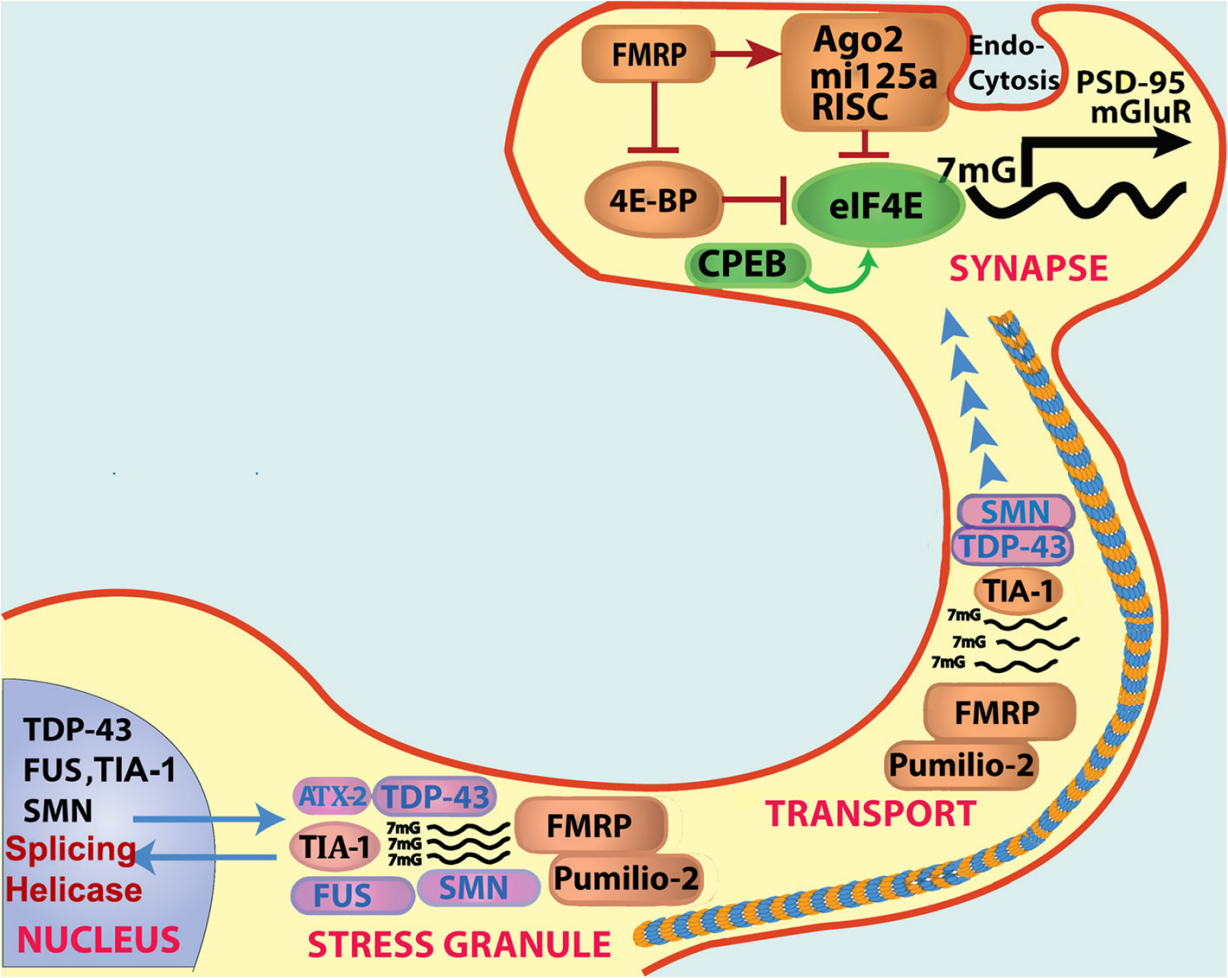
**Structure and functions of RNA binding proteins.** RNA binding proteins have dual sites of action. In the nucleus, many RNA binding proteins, such as TDP-43, SMN (SMN1 and 2), TIA-1 and FUS regulate mRNA splicing. RNA binding proteins are also present in the cytoplasm and neuronal arbors, where they regulate RNA transport, activity dependent protein synthesis and sequestration of unnecessary transcripts in response to stress. Each of the RNA binding proteins shown in the figure associate with stress granules. TIA-1, SMN and Pumillio-2 are important for trafficking of mRNA in axons and dendrites, which is mediated by microtubules (blue and mustard striped line) and molecular motors. At the synapse, different RNA binding proteins regulate activity dependent translation. Phosphorylation causes 4E-BP to dissociate from eIF4E, which initiates translation. FMRP inhibits this process; loss of FMRP expression (such as occurs in fragile X syndrome) leads to excessive synaptic protein synthesis and excessive dendritic spine density. In contrast, CPEB stimulates activity dependent translation in a process that might involve regulated protein aggregation [27]. Activity dependent protein synthesis is modulated by microRNA. For instance, miR125 regulates the synthesis of mGluR and PSD-95 [24]. miRNA are generated by action of the RISC complex and argonaute, which cleave precursors to generate the miRs. Adapted from Liu-Yesucevitz, et al. [10].

The aggregation processes characterizing the biology of Sup35 and RNA binding proteins differ from the conventional models of protein aggregation in that they subserve distinct biological functions and are reversible. In order to understand how this biology plays out, it is important to examine the functions of RNA binding proteins throughout the biological cycle of mRNA processing. The functions of RNA binding proteins can generally be divided into nuclear and cytoplasmic activities, each of which is the subject of very large fields of literature. In the nucleus RNA binding proteins regulate mRNA maturation, including splicing, RNA helicase activity, RNA polymerase elongation and nuclear export (Figure 3) [18]. In the cytoplasm RNA binding proteins regulate RNA transport, silencing, translation and degradation (Figure 3) [19]. These RNA binding proteins regulate transcript activity and distribution by forming RNA granules that are macromolecular complexes containing RNA binding proteins and mRNA transcripts consolidated to form granules through protein/protein interactions mediated by the glycine rich domains and protein/mRNA interactions mediated by RRMs [20]. RNA granules vary by molecular composition and function. RNA degradation is mediated by a type of RNA granule, termed the P-body [21]. Transport granules play important roles in neurons, where they move transcripts from the soma into the dendritic, and axonal, arbors [20]. RNA binding protein complexes also mediate the process of activity dependent protein synthesis, which is critical in all aspects of biology, but has attracted particularly strong attention at the synapse where it controls synaptic plasticity, habituation and memory [22]. The synaptic function of one RNA binding protein, Fragile X mental retardation protein (FMRP) has been studied extensively and is known to regulate dendritic sprouting [19,23,24]. RNA binding proteins also interface with the micro-RNA system, since both microRNA and RNA binding protein regulate protein synthesis [25,26]. The interaction of microRNA with RNA binding protein adds an additional layer of regulatory control.

## Stress granules and regulated protein aggregation

Stress granules (SGs) are another type of RNA granule that is generated in response to stressful conditions. Stressed cells need to produce cytoprotective proteins quickly. This is accomplished by formation of SGs, which shift RNA translation towards cytoprotective proteins [28]. As mentioned above, SGs sequester and silence non-essential transcripts, adapt local patterns of translation within the cell and sequester signaling molecules that regulate cell viability [19,29]. SGs also interact with other types of RNA granules. SGs can be seen adjacent to P-bodies, putatively transferring transcripts to the P bodies for degradation [30]. Neuronal transport granules, which facilitate activity dependent translational machinery at the synapse, can be converted to SGs. Thus, RNA granules interact with each other and can convert their functions depending on conditions.

The process of regulated protein aggregation that characterizes SG formation and allows RNA binding proteins to consolidate transcripts contrasts with vesicle formation, which is the other major cellular mechanism for consolidating molecules. Organelles such as the nucleus, mitochondria, peroxisomes, lysosomes and endosomes consolidate material by surrounding the concentrated target material with one or two lipid membranes. However, regulated protein aggregation achieves molecular consolidation using a process of reversible protein aggregation [31]. In cell culture, SGs form within minutes of a severe stress, and disappear 1 – 3 hrs after the stress is removed [31].

**Figure 4:**
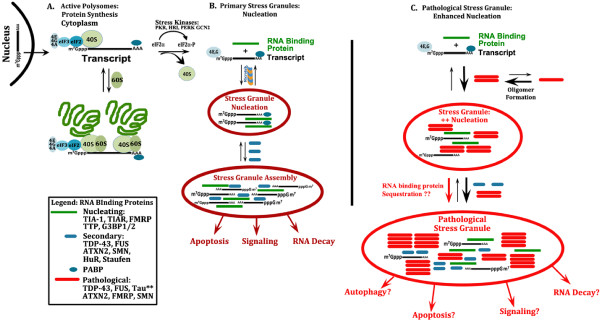
**Mechanism of normal and pathological stress granule formation.A**) In normal, physiological conditions, neurons synthesize specialized proteins from capped transcripts. The proteins eIF4A, E and G complex to form the eIF4F pre-initiation complex, which interacts with the ribosome (40S) as well as other translational regulators to synthesize proteins. Association with the 60S ribosome complex allows protein synthesis to begin. **B**) Stress leads to phosphorylation of eIF2α, dissociation of ribosomes and many of the translation initiation factors, leaving mRNA bound eIF4G and poly-A binding protein. Nucleating RNA binding proteins bind the free RNA and also form protein/protein complexes, which initiate stress granule formation. Once initiated, other RNA binding proteins bind to the mRNA and to the nucleating RNA binding proteins to increase the size and complexity of SGs. These SGs are rapidly reversible upon removal of the stress, however prolonged SG formation affects cell biology by interacting with biological systems regulating apoptosis, signaling and RNA decay. **C**) Pathological proteins, such as TDP-43, FUS and tau, have a strong tendency to form oligomers, and then fibrils. The consolidation of RNA binding proteins during SG formation might promote oligomerization by creating cellular domains with higher concentrations of these proteins. Conversely, the increased stability of oligomers and fibrils might serve as a nidus for SG formation, leading to over-active SG formation. Microtubule associated protein tau also participates in this process because it mislocates to the soma and dendritic arbor leading to interactions with SG proteins and potentially stimulating SG formation [41]. Tau also directly binds RNA [42].

The process of SG formation is best understood for pathways mediated by phosphorylation of eIF2α. Stressful conditions prompt phosphorylation of eIF2α at serine 51, which inhibits formation of a complex containing eIF2, GTP and tRNA_i_^met^[31]. Stress also induces translocation of many RNA binding proteins from the nucleus to the cytoplasm. During stressful conditions, capped mRNA remains bound to the pre-initiation complex, which contains the other elongation factor binding proteins EF-4A, E and G (Figure 4). This mRNA-protein complex is bound by eIF3, poly-A and nucleating RNA binding proteins, such as TIA-1, TIAR, tristetraprolin (TTP) or GTPase activating protein binding protein (G3BP), which bind the “naked” transcripts in the cytoplasm (Figure 4) [28]. The SGs are initially small, but increase in size as the RNA binding proteins consolidate by binding to each other through the glycine rich protein aggregation domains. This process of secondary maturation of SGs specifically containing G3BP is a prominent cytoplasmic function of TDP-43 [32]. Mutations in RNA binding proteins appear to increase their propensity to aggregate and to form SGs. For instance, disease-linked mutations of TDP-43, FUS and ataxin-2 promote aggregation, either by directly increasing the tendency of the protein to aggregate, or (for many FUS mutations) by preventing nuclear translocation [16,17,33-37]. The SG complex initially forms a structure that is conceptually analogous to a tree, with the glycine rich aggregation domains forming the core of the structure, and the mRNA bound to the RRMI, hanging off the RNA binding proteins. The complex of aggregated RNA binding proteins grows with time as other RNA binding proteins are recruited through binding to the associated transcripts and binding to the protein aggregation domains of other RNA binding proteins (Figure 4). Mature granules contain many RNA binding proteins recruited after the initial nucleation event. The combined role of protein-protein interactions and protein-RNA interactions presents an important methodological consideration for those investigating SG structure. Biochemical studies of RNA binding protein associations in SGs require pretreatment with RNAse to determine whether SG proteins are directly associated or associated due to mutual binding to mRNA transcripts [16]. Biochemical studies of SGs also demonstrate that the RNA binding proteins present in SGs are triton or SDS insoluble (depending on the protein studied, and the conditions inducing the SG), which is analogous to the biochemistry of proteins aggregates in many neurodegenerative diseases [6,16,17,28,31,38-40].

The importance of SGs for cytoprotection is highlighted by the effects of knockout of SG proteins, such as TIA-1, or inhibition of eIF2α phosphorylation, which render cells more vulnerable to acute stresses [43,44]. Conversely, inhibiting eIF2α dephosphorylation protects against some forms of stress [45]. The actual mechanisms by which SGs mediate protection, though, are poorly understood. For instance, conditions of endoplasmic reticulum stress lead to formation of SGs that inhibit cytoplasmic RNA translation, while preferentially retaining RNA translation in the endoplasmic reticulum [46]. Global shifts in RNA translation or RNA degradation are also observed with other stresses, with the exact pattern of RNA translation or degradation varying depending on the particular stress [47]. (The translational response of heat shock protein 70 (HSP70) to stress is nuanced. HSP70 synthesis is stimulated by expression of the pro-aggregation prion domain of TIA-1, but HSP70 synthesis is not stimulated by over-expression of full length TIA-1 [6]. Although the HSP70 transcript contains capped mRNA, HSP70 synthesis does not appear to occur preferentially during SG formation, and might actually be inhibited [48]. On the other hand, protein aggregation, such as those involved in degenerative diseases, stimulates synthesis of HSP70. These seeming contradictions highlight important areas of HSP70 biology that are incompletely understood.) The composition of SGs also varies greatly depending on the type of stress. Since RNA binding proteins all exhibit different RNA binding profiles, the corresponding pattern of RNA translation will vary with the stress. Thus, the changes in RNA translation occurring with stress reflect the biochemical cascades responding to the particular stress, the location of SG formation within the cell, and the pattern of RNA sequestration induced by the composition of the SGs.

Signaling regulates each step of the RNA maturation cycle, including synthesis, splicing, formation of granules (including stress granules), RNA granule transport and activity dependent RNA translation. For instance, phosphorylation of the translation initiation factor, (eIF2α) silences translation and is regulated by four different kinases: PERK, HRI, GCN2 and PKR. The regulation of PERK activity has been perhaps the most intensively studied, since PERK mediates stress of the endoplasmic reticulum [49,50]. The SG response is classically designed to cope with a transient stress, and rapidly resolves after the stress is removed [31]. GADD34 is a phosphatase that selectively dephosphorylates eIF2α [51]. Dephosphorylating eIF2α disperses SGs and stimulates RNA translation. SG formation is also regulated through eIF2α-independent pathways such as cleavage of tRNA by angiogenin or inhibition of ribosomal scanning [52,53]. Signaling cascades such as mTOR, S6 kinase, MAP kinases and Mnks regulate activity dependent translation [54]. The strong role that these signaling cascades play in regulating SG formation demonstrates that RNA translation generally, and SG formation specifically, are both highly regulated processes. Thus, signaling cascades regulate the process of RNA binding protein aggregation, reversibly controlling SG formation or dissolution.

The process of SG formation and dispersion can also be regulated by exogenous application of chemicals that inhibit RNA translation. Cycloheximide and emetine inhibit SG formation by interfering with RNA translation at the step of protein elongation in a manner that maintains the polysomes, which prevents free mRNA from accumulating in the cytoplasm [15]. In contrast, puromycin stimulates SG formation by causing premature chain termination at the ribosome, inducing disassembly of the polysome and producing free mRNA which are then bound by RNA binding proteins [15]. These chemicals are toxic, and thus not useful clinically, but compounds such as salubrinal can induce SG formation without much toxicity [45]. In addition, my laboratory is currently studying novel compounds that inhibit SG formation without toxicity.

The stress response is an integrated response, and this integration also applies to SG production. The RNA binding proteins, TIA-1, TIAR, TTP and G3BP nucleate SGs; a full list of nucleating RNA binding proteins is provided in other reviews, but these four RNA binding proteins are the most commonly examined [29]. Maturation of SGs leads to incorporation of multiple other proteins that regulate other stress response systems. Since SGs are designed to be transient, their formation appears to inhibit irreversible cellular events, such as apoptosis. The pro-apoptotic proteins RACK1, ROCK1 and TRAF2 are all sequestered in SGs, which inhibits the apoptotic response [55,56]. Sequestered RACK1 is unable to activate apoptosis mediated by MTK1, and TRAF2 is unable to activate apoptosis mediated by TNFα and NFκB. Binding of RSK2 and FAST kinase further inhibit apoptosis, and also promotes translational repression [57]. Signaling molecules, such as JNK, MKK7 and rhoA are also recruited to SGs, as are scaffold proteins known to regulate other signaling cascades, such as AKAP350A and WDR62, which regulate the responses to cAMP and oxidation, respectively [58,59].

Cytoskeletal machinery facilitates the coalescing of RNA binding proteins to make the SG. Histone deacetylase 6 (HDAC6) is required for this process; it deacetylates tubulin to reduce microtubule-dependent motility, which promotes consolidation of cellular complexes, such as autophagosomes, aggresomes and mitochondria targeted for mitophagy [60-62]. HDAC6 is also required for SG formation [60]. Interestingly, microtubule associated protein tau also regulates transport, classically inhibiting anterograde transport. Stressful conditions lead to tau phosphorylation and mislocalization to the soma and dendrite, where it comes into contact with RNA binding proteins associated with stress granules [63]. Our recent results suggest that tau might modulate SGs [38]. Tau biology is integrated with that of HDAC6, which regulates its turnover [64]. Mutations in dynein motor subunits, such as DLC2A, localize with SGs and are required for SG formation, just as they are required for other stress processes; these proteins couple SGs to the cytoskeleton and facilitate consolidation into a local region within the cell [60,65]. Dynactin subunit I and profilin, both of which regulate actin filaments, cause familial ALS; since the SG protein TDP-43 accumulates in ALS, it is tempting to speculate that these proteins also regulate SG formation [66-68]. Not surprisingly, many of the signaling cascades known to regulate HDAC6 and molecular motors also regulate SG formation, including JNK, MKK7 and rhoA [59,65,69]. This suggests the presence of a common group of proteins contributes to consolidation of many of the structures occurring in response to stress, such as SGs, autophagasomes and aggresomes. The multiple different biological processes that impact on SG formation further emphasizes that the SGs are highly regulated and highly integrated into the biological response to stress and RNA translation.

## SGs co-localize with insoluble protein aggregates in neurodegenerative diseases

The potential importance of SGs for neurodegenerative disease becomes apparent because the process of SG formation presents a biological pathway that could be vulnerable to the protein aggregates that accumulate in neurodegenerative disease. RNA binding proteins are a group of proteins that naturally form insoluble aggregates, yet the aggregated material can disperse and resolubilize [28]. RNA-protein SG complexes are sequestered as SDS-soluble, but triton insoluble protein aggregates [16]. Most, if not all, of the RNA binding protein linked to neurodegenerative diseases associate with SGs in cell culture. TDP-43, FUS, ataxin-2, SMN, optineurin and angiogenin have all been shown to co-localize with classic SG markers (TIA-1, TIAR and/or G3BP) in cells undergoing stress [16,33,34,37,70]. SG proteins such as TIA-1, eIF3 and poly-A binding protein, PABP also co-localize with neuropathology in brain tissue of subjects with AD, FTDP-17, FTLD-TDP and ALS, or animal models of these diseases [16,38]. In addition, SMN, huntingtin and PrP^sc^ associate with SGs and modulate SG formation in cultured cells [39,40,71]. Our studies of brain tissues from animal models of tauopathy indicate that the solubility of different SG proteins varies dramatically among particular SG proteins. Biochemical fractionation of proteins from these samples shows that RNA binding proteins such as TIA-1, TTP and G3BP form aggregates that are triton insoluble but sarkosyl soluble [38]. In contrast, pathological aggregates of tau or β-amyloid are highly insoluble [38]. Interestingly, TDP-43 and FUS also form highly insoluble inclusions [16,34]. Thus the proteins that form the most insoluble aggregates are those that have been shown to cause familial forms of neurodegenerative disease, including TDP-43, FUS and tau. Our studies (discussed below) also point to a close link between tau and SG biology. Parkinson’s disease is perhaps the outlier, because α-synuclein and Lewy bodies have not yet been associated with SG biology, but leucine rich repeat kinase 2 (LRRK2) is known to modulate RNA translation, which points to a link with SG biology [72]. These results suggest that disease-linked proteins form pathological aggregates that are often, if not always, co-localized with SG proteins.

The examples of AD and FTLD-17 are particularly striking because in both of these tauopathies, the load of SG positive inclusions that form is large, exhibiting a density that is equal to or greater than the load of neurofibrillary tangles [38]. SG proteins such as TIA-1 and TTP identify most neurofibrillary tangles, but also identify inclusions that appear to lack reactivity with antibodies to phospho-tau (e.g., PHF-1 or CP13) or conformationally altered tau (e.g., Alz-50 or MC1). A different SG protein, G3BP, identifies neurons that are predominantly negative for pathological tau protein [38]. The abundance of SGs in tauopathies suggests that the load of pathological inclusions is much greater than would be apparent by simply using markers of tau protein.

The co-localization of SGs with neuropathology in the human brain suggests shared mechanisms. I propose that the aggregation of pathological proteins (e.g., tau or TDP-43) stimulates SG formation, and formation of SGs accelerates aggregation of the pathological proteins (Figure 4). As mentioned above, disease-linked pathological proteins, such as tau and TDP-43, are known to exhibit a tendency to form stable, insoluble protein aggregates. The interaction between pathology of SGs is easy to envision for RNA binding proteins that exhibit disease-linked mutations, such as TDP-43, FUS, optineurin and angiogenin, each of which are linked to ALS. Disease linked mutations in these proteins increase the tendency of the protein to aggregate. In addition, the aggregates that form appear to be highly insoluble, highly stable complexes. Since these proteins normally exist in equilibrium between dispersed, soluble proteins and aggregated, insoluble complexes; aggregation-accelerating mutations would shift the equilibrium, leading to increased SG formation and formation of the stable, long-lived protein aggregates that we associate with disease pathology (Figure 4). The presence of more aggregates and/or aggregates with enhanced stability might also increase formation of mature SGs around the aggregates, much like the normal evolution of SGs. This process of SG growth is analogous to cross-seeding, and normally forms the basis of SG maturation. Pathological cross-seeding already has been shown in cell culture for polyglutamine rich proteins, such as huntingtin, and for stimulation of tau aggregation by α-synuclein [71,73]. This same process of accelerated cross-seeding might occur in the brain for other pathological proteins, more generally. The formation of long-lived, stable insoluble protein aggregates could shift the equilibrium of regulated protein aggregation towards aggregation, leading to accelerated, long-lived SG formation (Figure 4). These highly insoluble aggregates could also serve as a nidus for further aggregation of SGs, by binding with other RNA binding proteins and also binding RNA as part of the process of SG maturation. The result would be an overactive SG pathway.

Overactive SG formation in tauopathies might seem puzzling, since tau was not previously considered to be a SG protein. However, our studies using transfected SH-SY5Y cells demonstrate that SG formation stimulates formation of phosphorylated tau inclusions [38]. Even more strikingly, tau appears to stimulate SG formation [38]. The latter result suggests that tau actually contributes to the SG response, and might explain the strong link between tauopathy and SGs. Evaluation of tau biology suggests a mechanism for interaction with SGs. Tau expression is normally restricted to the axon, while the RNA translation machinery is more abundant in the soma and dendrites. However, stress stimulates tau phosphorylation and mislocalization to the soma and dendrite where it can interact with RNA binding proteins, as well as RNA, associated with SGs [41,63]. This interaction accelerates SG formation, and also might accelerate tau aggregation. The mechanism remains to be determined, but the building blocks underlying a putative mechanism are evident. Tau binds SG proteins, such as TIA-1 and TTP, which provides for a direct interaction between tau and SGs [38]. In addition, RNA is a known stimulus for tau aggregation in vitro [74], which makes it possible that the RNA associated with SGs might further promote tau aggregation. Thus, in the case of tauopathies, aggregation of tau protein stimulates SG formation, leading to enhanced SG formation, and SG formation might stimulate tau aggregation.

Whether the overactive SG formation is good or bad remains to be determined. The response of neurons to SG formation might be analogous to the responses to autophagy; some autophagy is needed for survival, but too much autophagy can be deleterious. Neurons require SGs for an effective stress response, but overactive, overly stable SG complexes could easily interfere with neuronal function by silencing transcripts and sequestering important proteins. Mutations associated with disease-linked proteins increase the aggregation propensity, which provides a direct mechanism for overactive SG formation. Chronic stressful diseases or environmental conditions might also stimulate overactive SG formation. For instance, the oxidative stress associated with aging, the trophic stress associated with diabetes or the physical stress associated with chronic traumatic encephalopathy all enhance SG formation creating the conditions for pathological aggregation [75-77].

The effects of modulating the protein synthesis/SG pathway were recently evaluated in an animal model of Creutzfeld Jacob disease, where pathological misfolding of PrP precipitates neurodegeneration. Mallucci and colleagues forced expression of GADD34 to reduce eIF2α phosphorylation, inhibit SG formation and stimulate protein synthesis [51]. This intervention reduced PrP-induced neurodegeneration. In contrast, salubrinal, which increases eIF2α phosphorylation, increased SG formation, inhibited protein synthesis, and accelerated neurodegeneration [51]. These results suggest that inhibiting the SG pathway and stimulating protein synthesis can inhibit PrP-mediated neurodegeneration. The discovery of over-active SG formation in other diseases raises the possibility that these pathways are over-active in multiple neurodegenerative diseases, and that pharmacotherapy targeting SG formation might be neuroprotective.

## Critical questions

SG biology provides a highly useful paradigm for understanding neurodegenerative diseases, but important questions related to SG biology remain to be investigated. The major questions can be divided into four areas:

1. *What are the consequences of stress granule persistence?* SGs are classically transient structures but in neurodegenerative diseases they become associated with pathological structures and appear to persist. The biological consequences of this persistence are not known. Persistent SGs might protect the neuron by acting as a sink for sequestering toxic oligomers. However, persistent SGs might also serve as a sink for sequestering RNA binding proteins, which would interfere with their normal function. In ALS, for instance, the pathology is notable for the loss of nuclear TDP-43 and FUS. It seems possible that these proteins might be absorbed and sequestered by stable SGs. For instance, a recent study compared the effects of TDP-43 and FUS knockdown, and identified a discrete number of very long, brain specific transcripts that are regulated in common, including parkin and neurexin 3 [78]. Misregulation of these genes might contribute to disease. Kinetics is another critical consideration. A small group of nucleating RNA binding proteins initiate SG formation, which then continue to grow and incorporate other RNA binding proteins. Persistence of SGs might allow other proteins to interact with SGs, but with delayed kinetics. This could lead to ubiquitination of SGs, interactions of SGs with the autophagic system or dysfunction of pro-apoptotic proteins [57].

2. *Does translational repression contribute to the pathophysiology of neurodegenerative diseases?* The role of the SG pathway in translational repression also raises inherent questions about the role of translation repression in the pathophysiology of disease; the ability of GADD34 to delay progression of PrP mediated degeneration emphasizes the potential significance for therapy of neurodegenerative diseases [51]. It seems likely that as we understand how to manipulate this highly regulated pathway, we will better understand the biology of neurons and possibly gain insight into pathways for intervention in neurodegenerative diseases by modulating protein translation.

3. *Do deficits in axonal and dendritic transport affect stress granule biology?* Multiple genes linked to transport are genetically implicated in neurodegenerative diseases. Since RNA transport represents a large part of extra-nuclear function of RNA binding proteins, it seems possible that the pathophysiology of the transport biology might be linked to the pathophysiology of RNA binding proteins. Tau protein, which is a major component of the pathology in AD and FTD, regulates microtubule stability [41]. Mutations in transport proteins profillin, kinensin, dynein and dynactin are all associated with motor neuron diseases [66,79-81]. Whether these mutations affect transport of mRNA to the synapse or SG formation remains an open question.

4. *α-Synuclein and Parkinson’s disease:* Most of the genes linked to Parkinson’s disease appear to be most important to the biology of organelles and vesicles: mitochondria, autophagy, mitophagy, lysosomal function and vesicular endocytosis [82]. The link between Parkinson’s disease, RNA binding proteins and regulated protein aggregation remains nebulous. Some recent studies point to links with RNA translation and regulated protein aggregation, but the studies are small in number. Mutations in TDP-43 have been identified in some cases of PD [83]. TDP-43 pathology is present in diffuse Lewy body disease, which provides some support suggesting the involvement of RNA binding proteins in synucleinopathies, but no such pathology has been reported for PD [84-86]. Some studies also implicate RNA translation in the pathophysiology of PD. Abeliovich’s group identified an association between the length of the 3’ UTR of α-synuclein and Parkinson’s disease [87]. Finally, LRRK2 is implicated in the regulation of RNA translation [72]. Each of these connections are interesting, but whether these impact on the process of regulated protein aggregation remains to be determined.

5. *What are the pathways that regulate aggregation and dis-aggregation?* While some of the pathways regulating SG formation are defined, it seems likely that other pathways will be identified. In addition, very little is known about the mechanisms that dis-aggregate SGs. The ability of SGs to rapidly disperse raises the specter of biochemical pathways that can disperse (at least some) protein aggregates quicker and more effectively than the classic pathways known for HSPs, the ubiquitin proteasomal system and autophagy. Identifying such pathways could be particularly important in the context of neurodegenerative diseases where SG formation might be over active.

## Conclusion

This review presents the concept of regulated protein aggregation. The idea that protein aggregation might be physiological and regulated presents a novel paradigm for the field of neurodegenerative research. The classic model of pathological protein aggregation is a process that occurs through random interaction of misfolded proteins. In this review, I point out that many of the proteins linked to neurodegenerative disease assume a conformation that favors aggregation as part of the normal biology of RNA granule formation generally, and SG formation specifically. This physiological aggregation becomes pathological when the pro-aggregation state is favored because of mutations, other disease processes or environmental conditions. Linkage of pathological protein aggregation to the normal biological process of regulated protein aggregation is important because RNA granule and SG formation are regulated by signaling cascades and are reversible. The role of biochemical pathways in regulating regulated protein aggregation presents potentially novel targets for pharmacotherapy of neurodegenerative disease.

## Abbreviations

AD: Alzheimer’s disease; Ago2: Argonaute-2, eukaryotic translation initiation factor 2C; ALS: Amyotrophic lateral sclerosis; ANG: Angiogenenin; ATX2: Ataxin-2; CPEB: Cytoplasmic polyadenylation element binding protein; 4E-BP: Eukaryotic translation initiation factor 4E-binding protein; eIF2, 3: Elongation initiation factor 2, 3; eIF4E: Elongation initiation factor 4E; FMRP: Fragile X mental retardation protein; FTDP-17: Frontotemporal dementia with parkinsonism, chromosome 17; FTLD-TDP: Frontotemporal lobar dementia with TDP-43; FUS: Fused in sarcoma; G3BP: GTPase activating protein binding protein; GCN2: GCN2 eIF2α kinase; eIF2α kinase 4; HDAC6: Histone deacetylase 6; HRI: Heme regulated initiation factor 2α kinase; eIF2α kinase 1; HSP70: Heat shock protein 70; HuR: Hu antigen R, ELAV-like protein 1; LRRK2: Leucine rich repeat kinase 2; 7mG: 7-methylguanosine; mGluR: Metabotropic glutamate receptor; mi125a: MicroRNA 125a; OPT: Optineurin; PABP: Poly-A binding protein; PERK: PRKR-like endoplasmic reticulum kinase; eIF2α kinase 3; PKR: P1/eIF-2A protein kinase; eIF2α kinase 2; PrP^sc^: Prion protein, pathological form; PSD95: Post-synaptic density protein 95; RISC: RNA induced silencing complex; RRM: RNA recognition motifs; SG: Stress granule; SMN: Survival of motor neuron; TDP-43: Tar DNA binding protein-43; TIA-1: T-intracellular antigen-1; TIAR: TIA1 cytotoxic granule-associated RNA binding protein-like 1; TTP: Tristetraprolin.

## Competing interests

BW has applied for a patent related to the application of stress granule biology to neurodegenerative disease.

## Authors’ contributions

BW prepared and wrote the manuscript.
